# Modus Operandi of Zymotic Agents

**Published:** 1884-03

**Authors:** W. W. Hipolite

**Affiliations:** De Vall’s Bluff, Ark.


### Selections.

### MODUS OPERANDI OF ZYMOTIC AGENTS.*

BY W. W. HIPOLITE, M.D., DE VALL'S BLUFF, ARK.

[St. Louis Courier of Medicine.]

"A little leaven leaveneth the whole lump."—Corinthians, chap. v., ver. 6.

Of all the deaths which occur annually in the United States, nearly one-half are caused by what are generally designated the zymotic diseases. Considering this large proportion, it is not surprising that the attention of the profession should be especially directed to an investigation into the etiology, symptomatology, modes of propagation, etc., of these diseases, as inquiries which are likely to yield results of the greatest scientific interest and practical importance. But notwithstanding the vast amount of labor that has been spent upon this department of medicine—and that, too, by men of the highest scientific acquirements, aided in their researches by all the modern appliances of art—it must be admitted that comparatively little is yet known of at least some of the diseases of this class; and more especially is this true as regards the *materies morborum* by which they are produced.

From the obvious fact that every effect is necessarily the result of the operation of some cause—or of a combination of causes—and that the relations which exist between cause and effect must hold good in medicine as in every other science, it is evident that morbific agents of some kind are essential to the development of these diseases. It may at once be admitted that our belief in most of these causative agents is dependent upon inferential proof; that, save perhaps in a few instances, these subtle poisons have never been detected by the acutest human sense—that no positive morbid material has been discovered. This objection may be good, and sufficient to prevent the assumption of their existence as an ascertained and demonstrable fact; but it does not destroy the value of

*Read before the State Medical Society of Arkansas, 1883.

the inferential truth. The fact that we cannot perceive a thing by our gross senses is no proof of its non-existence. With all our boasted faculties we are beings of very limited perceptions. We are placed in a universe between the extremes of infinite magnitude and infinite minutiæ, and can perceive but a few links in the chain of existence which extends from the infinite to us and from us to nothing. The appliances of art, it is true, have greatly extended the range of our perceptions. The telescope has brought near the distant, and enabled us to grasp the dimensions of the vast, while the microscope has equally extended the sphere of our observation in the other direction, and enabled us to pereeive the existence of the minute. Still there is one infinity of magnitude beyond the powers of the telescope, and one infinity of minuteness which no microscope can reveal.

It must not be forgotten that the evidence of chemistry itself is often purely inferential, as, for instance, when we fail to prove the existence of a chemical substance by obtaining it in a separate form. Besides isolating such substances, we recognize their presence by witnessing the reactions which they display with various tests. Now the human body forms the most appropriate testing-apparatus for these peculiar morbific agents, to which the term zymotic has been applied; and it is this alone which affords the means of judging of their *dynamical* character - a character which is of vastly more importance than a knowledge of their physical or chemical properties. In chemistry less is known of the essential properties of a substance by examining it in a separate state than when it is studied in connection with other bodies, where its relations to these bodies may be ascertained So it is with the zymotic poisons—if we could always obtain them in a separate state and could subject them to a chemical analysis, we should know much less of their most important properties than can be ascertained by studying their actions in the living system.

Having determined, therefore, that the zymotic diseases are dependent upon causes of *some* kind, a close examination enables us to observe that the causes of zymotic disease—whatever they may be —are very different from those by which other diseases, not zymotic, are produced. By watching their effects upon the system, we find that they are distinguished from the ordinary causes of disease by several striking peculiarities; but it is exceedingly difficult, and perhaps impossible, to determine their *essential nature.* The diseases which they produce cannot be referred to a modification of the sensible or appreciable conditions of the atmosphere, for they prevail in all climates, at all seasons, under every possible thermometrical, hygrometrical, barometrical and electrical variations; and they are distinct and specific in character, and preserve their identity under all these varying circumstances.

In the absence of any positive conclusions as to their nature, speculation has been busy in framing various hypotheses on this point to aid in a solution of the phenomena to which they give rise. Those which afford a ready explanation of most, if not all, of the phenomena of zymosis, and which possess a good degree of plausibility, are four in number, and it seems probable that the truth must be contained among them. It may be that one of them is true, or that all of them are so. Still, it is to be recollected that they are claimed as nothing more than theories, for they have not received sufficient proof to insure their acknowledgment as established truths. The following are the forms of matter of which the so-called zymotic poisons are supposed to consist, as respectively assumed by these several hypotheses, viz.:

1. They are supposed to consist of organic compounds in a peculiar state of decomposition, which multiply themselves like ferments.

2. They may consist of nucleated cells:

3. They may be made up of microscopic vegetable fungi, or

4. They are supposed to consist of animalcules.

As a discussion of all the above hypotheses would require more time than can with propriety be devoted to it here, I shall limit myself to the consideration of the first of them—not, however, meaning thereby to imply that the others may not also be true. It is not claimed that all epidemic diseases are produced by chemical agents, but that some of them may depend upon causes included under one of the other hypotheses. While, therefore, I shall present the claims of this as *one* of the causes of zymotic diseases, I do not mean to exclude the agency of the other forms of matter.

The term zymotic (from *zyme*, ferment) was originally confined in its application to a class of toxic agents which have an action upon the blood analogous to that which a ferment has upon fermentable compounds—the former causing the materials of the blood to resolve themselves into a poison like itself, by a process similar to that by which the elements of the latter arrange themselves into new forms, possessed of new properties. On the one hand, by a new arrangement with relation to each other, the elements that previously constituted the blood have become changed into a zymotic poison, while, on the other hand, the fermentable compound has been destroyed, and its place supplied by the new products of fermentation—products which contain the particles that originally made up the compound itself. If confined to its literal meaning, the term would be limited to those poisons which act in this way; but it is now generally made to include all epidemic diseases, and signifies that material specific poisons are the essential causes of these diseases. Before considering their *modus operandi*, we will premise a few observations.

We know there are substances which act by a so-called catalytic power, and that in some instances chemical changes may be directly and unquestionably traced to the operation of this power. The property upon which these results depend—the power by which they are produced—has been denominated the "catalytic force," and the action itself is called catalysis or catalytic action. Starch becomes changed into grape sugar when it is subjected to the action of ferments or diluted acids at a temperature below the boiling point of water. An intermediate change takes place, the starch being first converted into dextrine and the dextrine into grape sugar. The acid does not in any way unite with the other elements, but may be precipitated at the close of the operation without loss, and the sugar so produced correspond in weight with the starch originally employed. Sugar may be converted into alcohol and carbonic acid by the action of a ferment. The yeast or ferment used to bring on this decomposition appears to act simply by its presence or by catalysis, its elements not uniting with any of the resulting products. It simply converts the sugar into alcohol and carbonic acid without being itself at all changed. When, instead of a solution of sugar, the expressed juice of various fruits is employed, there is produced a large quantity of yeast, derived from the gluten or vegetable albumen of the substance in operation. This, in its turn, can be used to set up fermentation in other liquids. By the action of sulphuric acid, lignin or woody fibre may be converted into dextrine; this, by the continued aid of heat, is converted into grape sugar; sugar, by the action of yeast, gluten, and a variety of animal and vegetable matters, is converted into alcohol and carbonic acid. Here, then, is a series of results brought about by catalytic action, commencing with the transformation of the fibre of wood, and ending in the production of alcohol and carbonic acid. And the catalytic forces are not confined in their action to organic substances, for we also find numerous examples of their influence in Inorganic Chemistry. These examples will serve to show that various substances may produce active and extensive changes in certain compounds without being themselves at all changed.

Organic compounds generally are characterized by a great proneness to decomposition or metamorphosis; and this instability is especially marked in those compounds which contain nitrogen—for, in its relation to the other elements, this is the most remarkable element known. Many of the compounds which contain it cannot be kept more than a few hours, after they have ceased to be under the control of vital laws, without the commencement of decomposition; and when they have entered into this state, they acquire the properties of a ferment, and are capable of exciting similar metamorphosis in other compounds if placed in contact with them. In this transformation the elements of the ferment take no chemical share in the metamorphosis of the body acted upon, but only act by inducing a state of change. Its agency, therefore, does not consist in furnishing any *material* to be employed in the composition of the resulting products, but in the *propagation of force;* it is not a *material*, but a *dynamical* influence. The existence, or the non-existence, of nitrogen in fermentable compounds gives rise to their division into two groups, the azotized and the non-azotized, or those which contain this element as one of its constituents, and those which do not contain it. Those comprising the first variety have their particles re-arranged in such a manner as to form a substance having all the properties of the ferment originally employed, while those comprising the second variety, although susceptible of metamorphosis, are not resolved into substances capable of exciting a like change in other bodies. In other words, the ferment is reproduced in the azotized compounds, but is not reproduced in the non-azotized. Hence the potency of a ferment will, in a great measure, depend upon the presence of a nitrogenous substance in which it can excite the same change, and through which it can act upon other fermentable matter.

Now, the blood contains a large proportion of nitrogen, and it is well known that there is no other part of the organism which can be compared to this fluid with respect to the feeble resistance it offers to exterior influences. Its chemical affinities are constantly tending to its decomposition and to the re-arrangement of its constituent elements in different forms from those existing, but it is endowed with a vital force which so modifies and controls the play of these affinities as to prevent decomposition while in possession of its normal amount of this force. As soon, however, as the *vis vitalis* is diminished below a certain point, the chemical affinities are enabled to prevail over the vital affinities, and metamorphosis commences immediately; or if, in addition, there is the catalytic force of some zymotic poison to aid in overcoming the vital force, the same result may be brought about more readily. Liebig says: "The chemical force and the vital principle hold each other in such perfect equilibrium that every disturbance, however trifling, or from whatever cause it may proceed, effects a change in the blood."

In its normal state the blood is constantly undergoing alterations both in its chemical constitution and in its vital properties. It supplies to the tissues some of its most important elements, and at the same time it is made the vehicle of removal from these tissues of ingredients which have lost their vital endowments and are no longer in a state of combination that fits them for their offices in the animal economy. Hence the blood contains not only the materials for the regeneration of the tissues, but also the products of their decay, and the results of the constructive and the destructive operations so exactly balance each other that there is a remarkable uniformity of composition of this fluid at all times during health. But the blood is different in different parts of the system, for the various tissues and organs make it subservient to their requirements—thus diminishing certain of its constituents and adding others. But the assimilative and eliminative functions so regulate these abstractions and additions that there is the same uniformity of composition of the blood from the same part. Anything that interferes with these changes, or turns them in another direction, may become a cause of disease.

In disease this vital principle may become so far depressed as to be unable to resist the natural chemical affinities of the elements of the blood, and hence changes may go on to a greater or less extent, according as the restraining force is more or less depressed. The balance of vital and chemical affinities being unstable, and the elements themselves tending to arrange the constituents of the blood in different forms from those existing—unless prevented by sufficiently powerful vital affinities—it takes but a slight force to destroy the equilibrium and overturn the existing arrangement of the elements and forces, so that new compounds are produced. These changes continue until compounds that are more stable, under the circumstances, are produced from the materials set free by the destruction of those which previously existed; or they may go on till the elements which originally constituted the blood are either set free in their simple elementary forms, or in the form of compounds that have little or no disposition to change, unless acted upon by some external substance or force.

The zymotic poison—a decomposing organic molecule—has been introduced into the blood. Now, this molecule has the power of imparting changes of an analogous character to other molecules which surround it; these, in their turn, excite disease in their neighbors, and by a continuance of this process from particle to particle the entire mass of the circulating fluid becomes changed. According to Liebig, the manner in which the septic matter operates upon the surrounding materials to induce a state of change in them is this: The particles or molecules of the exciting body, being in a condition of change, and, therefore, of motion, communicate to the molecules of the body placed in contact with them an amount of motion sufficient to destroy the balance of the existing affinities—which in organic compounds is easily done, the chemical equilibrium being very unstable—and thus gives rise to a new play of affinities and the production of new compounds. It is said to act by "catalysis," or by the "action of presence," as it is sometimes called. If the catalytic force be greater than the resistance offered by the *vis vitalis*, the blood must pass into a decomposing condition.

Let us view this operation from another stand-point. Each organized structure has the power of assimilating or appropriating to its own nature organizable materials which may be presented to it. So long as the structure remains healthy, it converts the materials to its own healthy condition, but when it becomes diseased, the substances are no longer assimilated to its original healthy, but to its altered or diseased condition. Thus bone converts the organizable materials into bone, muscle into muscle, etc., and when these are in a diseased state, the material is appropriated to the continuance of this state. It is so with the blood. In its normal condition it converts the nutrient materials to its own healthy nature, but when in an abnormal state, this state is continued or extended. The zymotic poison, when introduced into the circulating mass, has, as it were, a stronger assimilating power than has the blood itself; hence it deprives the blood of the nutrient materials presented to it, and this is appropriated to the building up of the poison. Not only this, but the poison converts the materials already assimilated—the blood itself—to its own condition, as well as the substances not yet appropriated. Some of these agents have a stronger determination to run their course and multiply themselves than others have, those of the greatest power being capable of inducing these changes when the circulating fluid possesses its normal amount of vitality, while others, again, are not able to act without a previous diminution of this, unless it is low in any given instance. The *vis medicatrix naturæ* attempts to eliminate them from the system, and frequently this can be done before the poison is much extended, but in some instances the poison extends itself in spite of nature, and this, too, in the most healthy blood.

As soon as the nutritive fluid becomes contaminated, it extends its depressing influence to every part of the organism, and every function feels this influence—becoming deranged in consequence. The natural emunctories through which the poison should be eliminated become sluggish and do not remove so much of the poison and effete matters as they do in their normal condition. In consequence of this the blood becomes still further poisoned by the retained materials, and these supply a peculiarly favorable nidus for the further development of the poison. Soon, instead of being the *pabulum vitæ* of the whole system, the blood becomes the vehicle of destruction and death to every part of the organism.

As a zymotic poison has the power of multiplying itself in the system, it might be inferred that a small quantity would be just as efficient as a large quantity in inducing changes and causing disease; and, accordingly, it is found that, however minute the quantity introduced, the potency of some of these poisons is such that they will change the whole mass of the blood in a short time. At first a person might be led to suppose that this would go to support the doctrine of Hahnemann, for, it will be recollected, he makes the minuteness of the doses one of the leading points in his doctrine—and this is about the only peculiar character it possesses in the estimation of the people. But by looking at this point for a moment, it will be seen that this support is only apparent, not real. In the first place, I do not conceive it possible for matter to be so finely divided—so far attenuated—as he recommends in the preparation of his infinitesimal doses. The zymotic poison is supposed to be an *organic molecule*, and must, therefore, consist of several chemical elements, each of which is composed of elementary molecules or atoms. Each of these atoms would be larger than one of Hahnemann's doses of medicine. Secondly, it is not claimed by him or his followers that their medicines multiply themselves in the system; but this is the leading characteristic of the agents we are considering, their potency being solely due to their self-multiplication. It is as though a large amount were at first introduced, without being subsequently multiplied. Thirdly, to cure a zymotic disease we would not think of introducing another poison similar to the one that caused the disease. If it were possible for the one to chemically neutralize the other, we might do so; but we have no evidence that this is ever done. When thus introduced each specific poison would have its own specific influence, just as each article of the materia medica has its peculiar action; for the blood may be the seat of action of more than one of these at the same time, although this is not generally the case. But as we have wandered from our subject, let us return.

That in zymotic diseases there is a change in the blood from an admixture of foreign matters is evident from the following classes of facts:

1. The phenomena of zymosis are produced when putrid matters are injected into the veins of animals.

2. They are produced by the entrance of this matter into the system in any other way, as by inhalation, etc.

3. These diseases can be produced by inoculation with their specific poisons, as in small-pox, etc.

4. Observation shows that in zymosis the blood is in a state of partial dissolution.

All these facts are so familiar to every physician of experience that it would be superfluous for me to dilate upon them here. One of the strongest arguments in favor of the zymotic theory of epidemic diseases lies in the fact that all those conditions which most favor the reception and propagation of the zymotic poisons are conditions in which there is an undue accumulation of effete azotized matters in the blood in a state of retrograde metamorphosis. This matter afford the best possible nidus for the further multiplication of these poisons; for it has lost its vital endowments, and is already in a state of change from the operation of ordinary chemical laws. In this condition the zymotic agent finds little difficulty in setting on foot those changes which are to result in the new generation of a like agent. And it has been found that this state of the blood is the most common and the most effectual predisposing cause to epidemic diseases which can exist; for there is present that peculiar soil, if I may be allowed the comparison, which is most rich in those elements required for the peculiar growths we are considering; and of all substances, these are most readily assimilated by the new growths. Thus, in those places which have the greatest accumulation of filth, you find the greatest mortality among the inhabitants during the prevalence of epidemics; and the rate of this mortality is in exact proportion to the uncleanliness, other things being equal. And so it is with individuals—those who have the greatest accumulation of decomposing matter in the blood are the ones who are soonest attacked by disease and removed by death upon the accession of an epidemic.

Dr. Carpenter arranges the generally recognized predisposing causes of zymotic diseases into three classes, viz.:

1. Those which tend to introduce into the system decomposing matter that has been generated in some external source, such as putrescent food, air charged with miasmatic emanations, etc.

2. Those which occasion an increased production of decomposing matter in the system itself; this increased production arises from any unusual source of degeneration of the tissues within the body, such as presents itself in the puerperal state, or as a consequence of severe muscular exertion, etc.

3. Those which obstruct the elimination of the decomposing matters normally or excessively generated within the system, or abnormally introduced into it from without. Among these causes are an insufficient supply of air, an elevated temperature, etc.

He goes on to show that each and all of these causes tend to the production of the very same condition; and this condition is "an accumulation of disintegrating azotized compounds, in a state of change, in the circulating current." He fully substantiates this view, and clearly proves that the zymotic poisons owe their potentiality to the condition in which they find their patient, and that many of them fail in producing their characteristic effects where this impure condition of the blood is not present. I have not the time, nor would it be necessary if I had, to present the facts and arguments which he has adduced in support of this position; but I think their existence will be apparent to every observing mind.

Heretofore we have only spoken of the zymotic poisons as being reinforced by new generation in the blood; but, it may be asked, do not some of them multiply themselves in the atmosphere, or externally to the body, and independently of it? Undoubtedly they are sometimes produced in this way, and it is readily understood how this might be done, for if they meet with organic matters susceptible of this transformation—other circumstances being favorable—they excite a change in them, just as they excite a change in the blood, by deranging the equilibrium of the chemical affinities. We see no reason why they cannot as well be generated without as within the body when the necessary conditions exist. It may be true, however, that the circulating fluid is generally the seat of these metamorphoses, because it is here that the requisite conditions are most likely to exist.

The decision of the question of the "contagious" or "non-contagious" character of any particular disease of this class rests upon a knowledge of the place where its specific poison is reinforced by new generation, whether this be within or without the body. As generally employed, the term "contagion" is loosely used, no precise and definite meaning being attached to it. Noah Webster gives a better definition of the term than will be found in most medical works. He says: "The words contagion and infection are frequently confounded. The proper distinction between them is this: contagion is the virus or effluvia generated in a diseased body, and capable of producing the specific disease in a healthy body by contact or otherwise; infection is anything that taints or corrupts, hence it includes contagion and any other morbid, noxious matter which may excite disease in a healthy body." A strictly contagious disease, then, is one, the specific poison of which is generated in the bodies of persons affected; and such a poison may be communicated to other persons in various ways, as by contact, fomites, etc. Those zymotic diseases, on the contrary, which result from causes generated exterior to the body, and are not capable of self-multiplication when introduced into it, are more properly denominated infectious. Such germs may be carried by men, goods, ships, etc., or may be scattered by the winds, to germinate whenever the requisite special conditions are found. In other words, the germs of contagious diseases are produced *in* the body, while the germs productive of infectious or non-contagious diseases are elaborated and re-elaborated *out* of the body and independent of its agency. "One is the product of *person*, the other of *place*."

